# Post-interventional infectious complications in percutaneous transabdominal lymphatic interventions: an observational study

**DOI:** 10.1038/s41598-023-42197-9

**Published:** 2023-10-17

**Authors:** Claus Christian Pieper, Sergej Geiger, Patrick Kupczyk, Julian A. Luetkens, Thomas Köster, Ulrike I. Attenberger, Hans Heinz Schild

**Affiliations:** 1 Division for Minimally-invasive Lymph Vessel Therapy, Department of Diagnostic and Interventional Radiology, University of Bonn, Bonn, Germany; 2 Department of Radiology, University of Bonn, Venusberg-Campus 1, 53105 Bonn, Germany

**Keywords:** Infection, Outcomes research, Antimicrobial therapy

## Abstract

The purpose of this retrospective study was to evaluate the occurrence of infectious complications and inflammatory reactions after transabdominal lymphatic-interventions. 63 lymphatic-interventions were performed in 60 patients (male/female: 35/25; mean age 56 [9–85] years) [chylothorax n = 48, chylous ascites n = 7, combined chylothorax/chylous ascites n = 5]. Post-interventional clinical course and laboratory findings were analyzed in the whole cohort as well as subgroups without (group A; n = 35) and with peri-interventional antibiotics (group B; n = 25) (pneumonia n = 16, drainage-catheter inflammation n = 5, colitis n = 1, cystitis n = 1, transcolonic-access n = 2). No septic complications associated with the intervention occurred. Leucocytes increased significantly, peaking on post-interventional day-1 (8.6 ± 3.9 × 10^6^ cells/mL vs. 9.8 ± 4.7 × 10^6^ cells/mL; p = 0.009) and decreased thereafter (day-10: 7.3 ± 2.7 × 10^6^ cells/mL, p = 0.005). CRP-values were pathological in 89.5% of patients already at baseline (40.1 ± 63.9 mg/L) and increased significant on day-3 (77.0 ± 78.8 mg/L, p < 0.001). Values decreased thereafter (day-15: 25.3 ± 34.4 mg/L, p = 0.04). In subgroup B, 13/25 patients had febrile episodes post-interventionally (pneumonia n = 11, cystitis n = 1, drainage-catheter inflammation n = 1). One patient developed biliary peritonitis despite continued antibiotics and underwent cholecystectomy. Baseline leucocytes and CRP-levels were higher in group B than A, but with comparable post-interventional profiles. Clinically relevant infectious complications associated with transabdominal lymphatic-interventions are rare irrespective of peri-interventional antibiotic use. Post-interventional elevation of leucocytes and CRP are observed with normalization over 10–15 days.

## Introduction

Lymphatic interventions are increasingly employed in the treatment of central lymphatic pathologies; thoracic duct embolization has meanwhile been accepted as an alternative to surgical treatment^1–3^. Although sometimes the thoracic duct may be cannulated through a retrograde transvenous or transcervical route^4,5^, the majority of interventions are performed through a transabdominal access^3,6–9^. With such an access path, a variety of organs including gastrointestinal structures may be transgressed during intervention^10^, raising concerns of infectious complications. Therefore antibiotic coverage has been recommended for lymphatic interventions^1,9,11–14^, although the rates of infectious complications and (patho)-physiological changes in inflammatory parameters have never been systematically evaluated. The aim of this retrospective study was therefore to report the occurrence of infectious complications and laboratory changes after transabdominal lymphatic interventions, in patients without peri-interventional antibiotic coverage and those with antibiotics due to intervention-unrelated reasons^7,15^.

## Materials and methods

### Patient selection

 Consecutive patients were included into the study based on inclusion criteria summarized in Table 1^11^.

**Table 1:** Patient inclusion criteria.

Inclusion criteria
Presence of chylous effusions
Proven by laboratory results of drained fluid (triglycerides > 110 mg/dL) Refractory to conservative treatment (at least 10 days of medium-chain triglyceride (MCT) diet and/or parenteral nutrition)
Lymphatic intervention to treat a central lymphatic pathology
At our institution Using a transabdominal access Between 2010 and 2018

### Interventional technique and antibiotics

**Figure 1:**
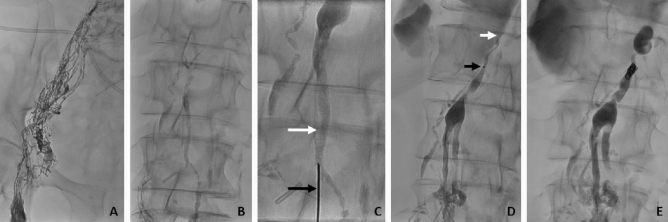
71-year-old woman referred for interventional treatment of bilateral, intractable chylothorax after oesophagectomy due to oesophageal cancer with drainage volumes > 2000 ml/day. X-ray lymphangiography was performed through an inguinal nodal access (**A**) leading to opacification of retroperitoneal lymphatics (**B**). (**C**) Fluroscopically-guided transabdominal puncture of the left lumbar trunk (white arrow) below the cisterna chyli was performed with a fine-needle (black arrow). After successful access, a micro-catheter (catheter tip: short black arrow) was advanced into the thoracic duct over a micro-wire (D) and transcatheter ductography showed transection (short white arrow) of the thoracic duct with extravasation into the thorax. After embolization with micro-coils and liquid embolic (**E**) the chylothroax resolved completely.

Procedures were performed by the same interventional radiologists (H.H.S. and/or C.C.P. with over 40 years and 10 years of interventional experience). Interventional techniques have been described in detail elsewhere^3,7,12,15,16^. X-ray lymphangiography was performed through an inguinal nodal access to identify the underlying lymphatic pathology and a target for transabdominal puncture (lymph vessels/nodes)^16^. A 21G fine-needle (Cook Medical, Bloomington, USA) was used for fluoroscopically-guided puncture of retroperitoneal lymph vessels/nodes. For localized abdominal pathologies, glue embolization was performed directly through the needle after access to a lymph node close to the pathology. For lymph vessel catheterization a 2.7F-microcatheter (Renegade, Boston Scientific, Marlborough, USA) was introduced over a guide-wire (Transend, Stryker Neurovascular, Fremont, USA). The catheter was advanced into the target vessels as selectively as possible. Embolization was performed using coils and/or glue (Histoacryl; Braun, Melsungen, Germany) mixed with iodized oil (Lipiodol; Guerbet, Sulzbach, Germany)^17^ (Fig. 1).

Patients did not receive routine pre-/peri-interventional antibiotics. Antibiotic therapy for other causes started prior to the intervention was continued peri-/post-interventionally as clinically indicated.

**Figure 2:**
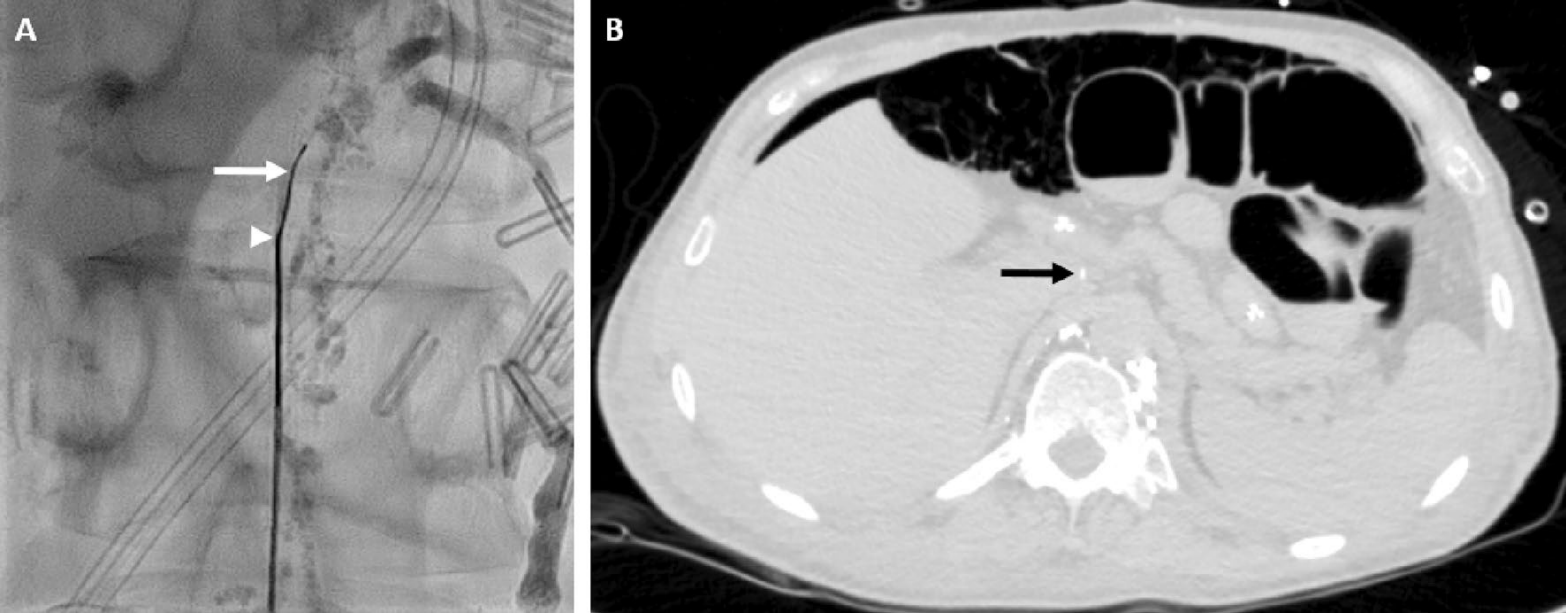
55-year-old man with intractable chylothorax after oesophagectomy. The intra-interventional fluoroscopic image (**A**) shows distension of the colon in the upper abdomen so that transcolonic puncture was necessary to access the target retroperitoneal lymphatics (tip of fine needle: white arrow head; micro-wire introduced into lymph vessel: white arrow). Post-interventional CT (**B**) showed free air in the abdomen after transcolonic puncture. The puncture tract can faintly be identified (black arrow). Antibiotics were started immediately after the intervention. The post-interventional course was uneventful without complications from transcolonic puncture. Chylous drainage ceased immediately after the intervention.

If during a procedure a transcolonic access was necessary, antibiotic coverage with cefuroxim was started (Fig. 2).

### Data analysis and definitions

Data sources included electronic patient charts and the radiology database/picture archiving system. Analysis included clinical demographics (sex, age, reason/location of lymphatic leakage, comorbidities), procedural (access route and material used for access, embolization material) and post-procedural data (post-interventional course, complications, survival).

Clinical success was defined as resolution of chylous leakage after the intervention. Clinical adverse events were recorded and graded according to the Common Terminology Criteria for Adverse Events (CTCAE, Version 5) with special focus on infectious complications associated with the intervention. Febrile episodes in the days after the intervention were noted.

Laboratory examinations of leucocytes and C-reactive protein (CRP) were ordered pre-interventionally (baseline), on day 1, 3, 10 and 15 after the intervention (depending on the duration of patients’ hospitalization).

Changes in laboratory values both within and outside the normal ranges valid for our laboratory were recorded (normal ranges: leucocytes: 3.6–10.5 × 10^6^ cells/mL; CRP: < 3 mg/L). Values outside the normal ranges were classified as pathological.

### Statistical analysis

Statistical analyses were performed using SPSS, version 27.0 (IBM, Armonk, NY). Descriptive statistics were done for patient characteristics and procedural parameters. Changes in laboratory parameters were evaluated by Student’s t-tests after evaluation of normal distribution using QQ-plots.

Additionally, subgroup analyses were performed according to the administration of antibiotics:

- Group A *without* pre-/peri-interventional antibiotics and
- Group B *with* pre-/peri-interventional antibiotics.

Group B was sub-stratified into patients, who received antibiotics already pre-interventionally due to other causes and patients, who started antibiotics during/after the intervention. Group comparisons of baseline patient characteristics and laboratory findings were performed using t-tests or Fisher’s exact test. Survival analysis for these subgroups was performed using the Kaplan–Meier method with Chi-square-test for statistical significance. Values < 0.05 were regarded as statistically significant.

### Ethical approval and informed consent

The presented study was approved by the institutional review board of the University of Bonn and hence all methods were performed in compliance with the ethical standards set in the 1964 Declaration of Helsinki as well as its later amendments. Written informed consent was waived.

## Results

### Patients

60 patients (35 male, 25 female; mean age 55.8 [9–85] years) fulfilled the inclusion criteria. 48/60 patients (80%) suffered from a chylothorax, 7/60 (11.8%) from chylous ascites and 5/60 (8.3%) from combined chylothorax/chylous ascites. 63 transabdominal lymphatic interventions were performed as 3 patients underwent a second intervention due to only partial clinical success of the first intervention. 2/63 interventions also included a direct transcervical access.

58/63 interventions (92.1%) were performed through a micro-catheter after lymph vessel puncture while the remaining 5/63 (7.9%) involved interstitial lymph node embolization through the puncture needle. In 56/63 (88.9%) embolization was achieved with coils in combination with glue and in 7 (11.1%) with glue alone.

Interventional treatment was clinically successful in 56/60 patients (93.3%). 4/60 patients (6.7%) continued to have effusions. Post-interventional clinical follow-up was available for all patients with a mean follow-up time of 820 days (range 44–4036 days). Detailed patient characteristics are given in Table 2.

**Table 2:** Patient characteristics.

	All patients	Group A (no antibiotics)	Group B (peri-interventional antibiotics, all)	Group B (pre-interventional antibiotics)	Group B (post-interventional antibiotics)	p-value
Number of patients	60	35	25	16	9	
Male: female	35:25	17:18	18:7	11:5	7:2	0.06
Mean age (SD; range)	55.8 (± 18.2; range 9–85) years	52.6 (± 19.5; range 9–85) years	60.4 (± 15.4; range 19–85) years	59.2 (± 15.3; range 19–76) years	62.5 (± 16.3; range 31–85) years	0.1
Indication for lymphatic intervention						0.053
Chylothorax	48	26	22	14	8	
Chylous ascites	7	7	–	–	–	
Combined chylothorax /chylous ascites	5	2	3	2	1	
Comorbidities						0.054
Cardiovascular disease	14	5	9	6	3	
Malignancy	33	20	13	8	5	
Liver cirrhosis	2	–	2	2	–	
Others	12	6	6	6	–	
Indication for antibiotics						
Pneumonia	16		16	12	4	
Drainage catheter inflammation	5		5	3	2	
Colitis	1		1	1		
Cystitis	1		1		1	
Transcolonic puncture	2		2		2	

*SD* standard deviation; p-value given for comparison between subgroups A and B.

### Antibiotics

35/60 patients (58.3%) with 37 interventions did not receive pre- or peri-interventional antibiotics (group A). 25/60 patients (41.7%) with 26 interventions received antibiotics (group B). These patients were further subdivided:

- 16/25 (64%) patients already were on antibiotics pre-interventionally [pneumonia (n = 12), inflammation of indwelling drainage catheter (n = 3), colitis (n = 1)].
- In 7/25 (28%) cases antibiotics were started post-interventionally [invasive ventilation-associated pneumonia (n = 4), inflammation of preexisting drainage catheters (n = 2; 1 thoracic, 1 abdominal), and cystitis (n = 1)]. In these cases antibiotic therapy was started on the day after the intervention in all cases.
- In two patients antibiotics were started peri-interventionally (immediately after the intervention) due to an unavoidable transcolonic puncture.

Antibiotics administered in group B were ampicillin/sulbactam (n = 13; 52%), cefuroxim (n = 6; 24%), piperacillin/tazobactam (n = 3; 12%), ciprofloxacin (n = 1; 4%), meropenem (n = 1; 4%) and meropenem/vancomycin/metronidazol (n = 1; 4%).

Comparing group A and B showed that less female patients received antibiotics and that all patients treated for chylous ascites did not receive antibiotics. However, overall baseline group differences did not reach statistical significance concerning gender, age, indications for a lymphatic intervention or comorbidities (Table 2). The latter were mainly oncological and cardiovascular in nature in both groups.

### Laboratory findings (Table 2; Figs. 3 and 4)

**Figure 3:**
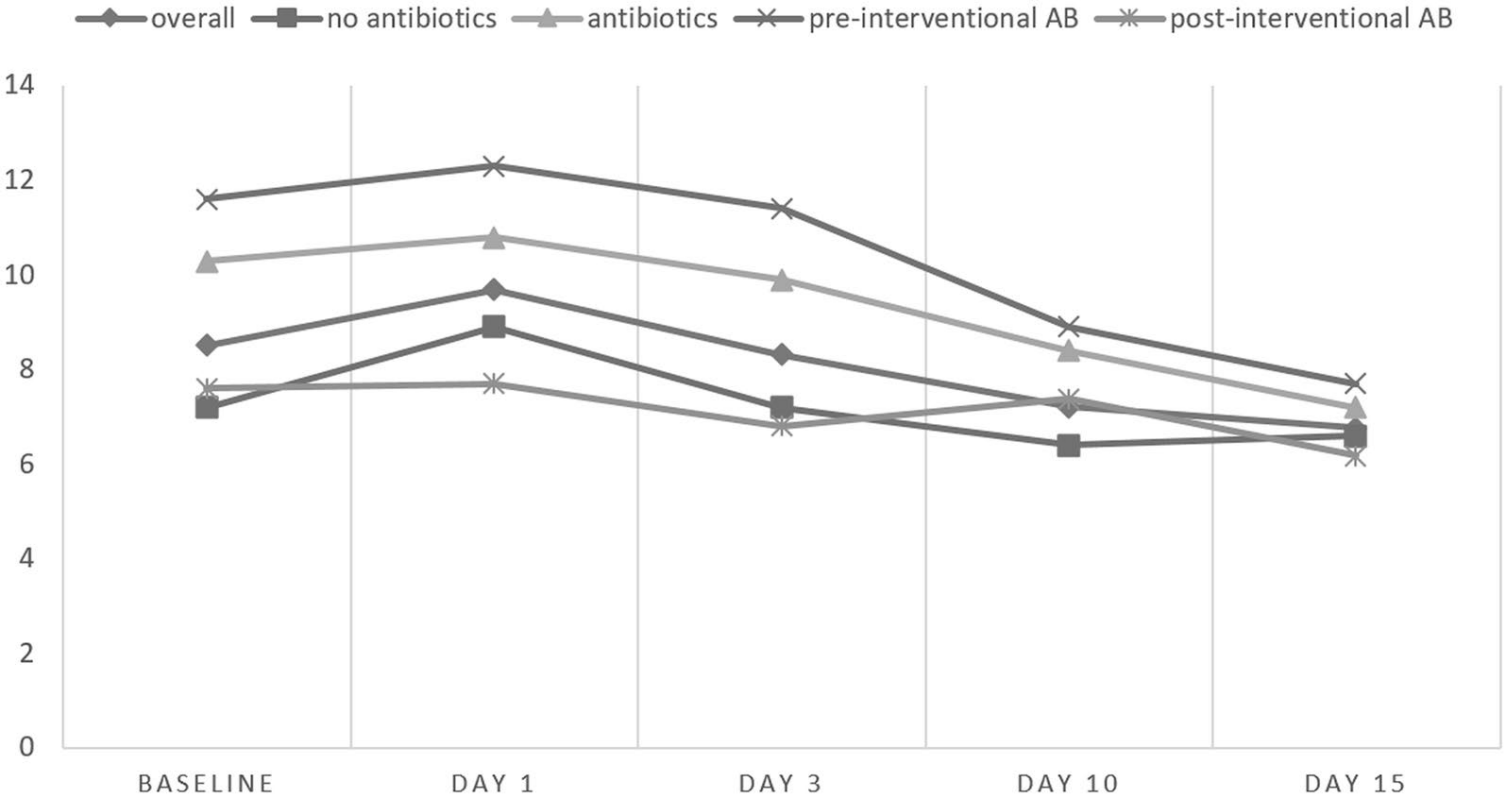
Mean leucocytes. The graphs display mean leucocyte levels [× 10^6^ cells/mL] at baseline as well as on day 1, 3, 10 and 15 after transabdominal intervention. Data are given for the whole cohort as well as stratified by application of pre-/peri-interventional antibiotics.

**Figure 4:**
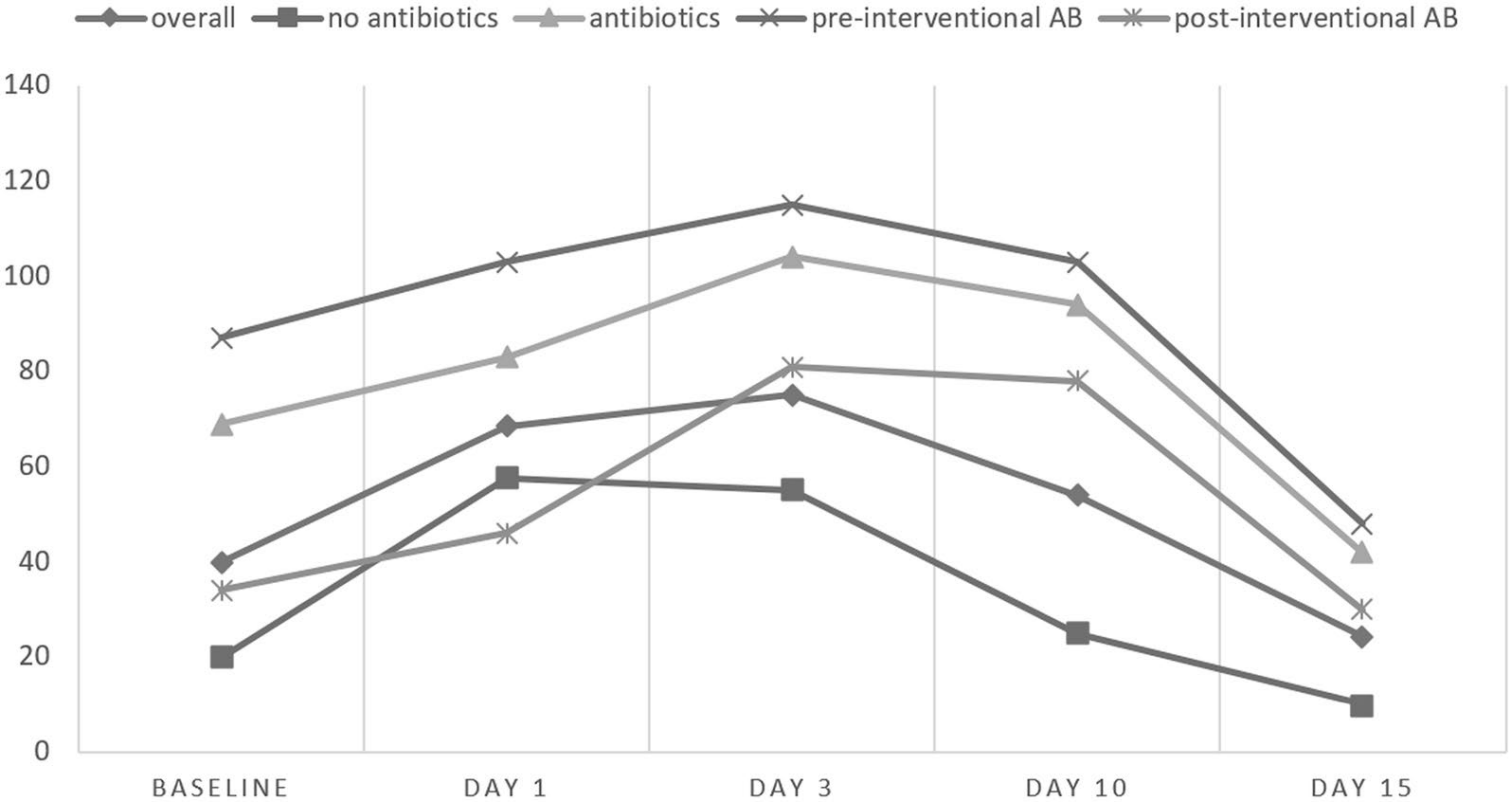
Mean C-reactive protein levels. The graphs display mean CRP-levels [mg/L] at baseline as well as on day 1, 3, 10 and 15 after transabdominal intervention. Data are given for the whole cohort as well as stratified by application of pre-/peri-interventional antibiotics.

Laboratory data were available in 57/60 patients (95%) at baseline, day 1 and day 3, in 56/60 (93.3%) at day 10 and in 48/60 (80%) at day 15 (Table 3).

**Table 3:** Laboratory findings.

	Baseline		Day 1			Day 3			Day 10			Day 15		
	Mean ± SD (range)	Pathological values among all tests	Mean ± SD (range)	Pathological values among all tests	p	Mean ± SD (range)	Pathological values among all tests	p	Mean ± SD (range)	Pathological values among all tests	p	Mean ± SD (range)	Pathological values among all tests	p
Leucocytes	8.6 ± 3.9; (2.5–20.5)	12/57 (21.1%)	9.8 ± 4.7 (1.5–23.5)	21/57 (36.8%)	**0.007**	8.5 ± 5.0 (1.6–35.0)	16/57 (28.1%)	0.804	7.3 ± 2.7 (1.3–17.1)	6/56 (10.7%)	**0.005**	6.8 ± 2.2 (1.8–12.5)	5/48 (10.4%)	** < 0.001**
Group A	7.4 ± 2.7 (2.6–14.5)	4/33 (12.1%)	9.2 ± 4.2 (3.4–21.7)	11/33 (33.3%)	**0.009**	7.4 ± 3.4 (2.9–15.7)	8/33 (24.2%)	0.931	6.4 ± 2.1 (2.7–11.0)	1/32 (3.1%)	**0.038**	6.5 ± 1.8 (4.2–10.8)	3/26 (11.5%)	**0.018**
Group B	10.3 ± 4.7 (2.5–20.5)	8/24 (33.3%)	10.8 ± 5.2 (1.5–23.5)	10/24 (41.7%)	0.380	9.9 ± 6.4 (1.6–35.0)	8/24 (33.3%)	0.710	8.4 ± 3.1 (1.3–17.1)	5/24 (33.3%)	**0.047**	7.2 ± 2.6 (1.8–12.5)	2/22 (9.1%)	**0.004**
Pre-interventional antibiotics	11.6 ± 4.6 (4.1–20.5)	6/16 (37.5%)	12.3 ± 5.2 (6.2–23.5)	8/16 (50.0%)	0.427	11.4 ± 7.1 (4.6–35.0)	7/16 (43.8%)	0.889	8.9 ± 2.3 (4.7–12.5)	4/16 (25.0%)	**0.042**	7.7 ± 2.0 (4.4–12.5)	1/15 (6.7%)	**0.015**
Post-interventional antibiotics	7.6 ± 3.9 (2.5–12.5)	2/8 (25.0%)	7.8 ± 4.0 (1.5–13.1)	2/8 (25.0%)	0.633	6.9 ± 3.1 (1.6–12.1)	1/8 (12.5%)	0.255	7.4 ± 4.6 (1.3–17.1)	1/8 (12.5%)	0.824	6.2 ± 3.5 (1.8–11.3)	1/7 (14.3%)	**0.025**
CRP	40.1 ± 63.9 (0.3–306.0)	51/57 (89.5%)	69.4 ± 74.9 (0.3–317.8)	53/57 (93.0%)	** < 0.001**	77.0 ± 78.8 (1.4–314.0)	55/57 (96.5%)	** < 0.001**	55.4 ± 67.6 (1.5–335.0)	52/56 (92.9%)	0.103	25.3 ± 34.4 (1.3–168.0)	43/48 (89.6%)	**0.040**
Group A	18.8 ± 14.5 (0.3–57.6)	28/33 (84.8%)	58.9 ± 59.6 (0.3–220.0)	29/33 (87.9%)	** < 0.001**	57.2 ± 63.5 (1.4–258.0)	31/33 (93.9%)	**0.001**	25.8 ± 24.4 (1.5–93.1)	28/33 (84.8%)	0.107	10.9 ± 9.4 (1.3–41.0)	22/26 (84.6%)	**0.010**
Group B	69.1 ± 90.1 (3.0–306.0)	23/24 (95.8%)	83.9 ± 91.3 (4.3–317.8)	24/24 (100%)	0.214	104.1 ± 90.3 (6.7–314.0)	24/24 (100%)	**0.049**	94.8 ± 85.4 (6.8–335.0)	24/24 (100%)	0.223	42.4 ± 44.5 (2.0–168.0)	21/22 (95.5%)	0.114
Pre-interventional antibiotics	86.8 ± 102.3 (3.8–306.0)	16/16 (100%)	103.0 ± 102.4 (4.3–317.8)	16/16 (100%)	0.332	115.7 ± 82.6 (15.3–304.0)	16/16 (100%)	0.100	103.1 ± 95.9 (7.6–335.0)	16/16 (100%)	0.568	48.4 ± 51.6 (2.0–168.0)	14/15 (93.3%)	0.131
Post-interventional antibiotics	34.1 ± 46.3 (3.0–141.0)	7/8 (87.5%)	45.8 ± 49.2 (5.9–141.0)	8/8 (100%)	0.410	81.0 ± 106.1 (6.7–314.0)	8/8 (100%)	0.275	78.3 ± 61.8 (6.8–183.0)	8/8 (100%)	0.141	29.6 ± 21.2 (6.0–64.0)	7/7 (100%)	0.696

*Group A* no antibiotics, *Group B* peri-interventional antibiotic use; p-values are given for statistical comparison (Student’s t-test) of follow-up laboratory values with the baseline. Statistically significant changes (p < 0.05) are given in bold.

#### Leucocytes

Baseline leucocytes were pathologically elevated in 12/57 (21.1%) patients (8.6 ± 3.9 × 10^6^ cells/mL); a significant increase was seen on day 1 after intervention (9.8 ± 4.7 × 10^6^ cells/mL; p = 0.009; pathological values in 21/57 cases [36.8%]). Leucocytes reached their peak on day 1, decreased thereafter and were significantly lower on day 10 and 15 compared to baseline (day 10: 7.3 ± 2.7 × 10^6^ cells/mL, p = 0.005; pathological in 6/56 [10.7%]).

##### Leukocytes in group A

The same pattern was seen in group A with a significant increase in leucocytes on day 1 without clinical signs of infection (from 7.4 ± 2.7 × 10^6^ cells/mL; pathological in 4/33 [12.1%] to 9.2 ± 4.2 × 10^6^ cells/mL; pathological in 11/33 [33.3%]; p = 0.009). Thereafter a significant decrease with normalization of values was seen (day 10: 6.4 ± 2.1 × 10^6^ cells/mL, p = 0.038; pathological in 1/32 [3.1%]).

##### Leucocytes in group B

In group B leucocytes were already significantly higher at baseline compared to group A (10.3 ± 4.7 × 10^6^ cells/mL vs. 7.4 ± 2.7 × 10^6^ cells/mL; p = 0.01) with pathological values already in 8/24 patients (33.3%). Values showed a further slight increase on day 1 (10.8 ± 5.2 × 10^6^ cells/mL; pathological in 10/24 [41.7%]). Leucocytes also decreased significantly during the follow-up period.

#### CRP

CRP-values were available for 57/60 patients and were pathological in 51/57 patients (89.5%) already at baseline (40.1 ± 63.9 mg/L). There was a further significant increase on day 1 and day 3 (69.4 ± 74.9 mg/L, p < 0.001 and 77.0 ± 78.8 mg/L, p < 0.001). Compared to leucocytes, CRP usually reached its peak later (on day 3). Values decreased thereafter to a mean of 25.3 ± 34.4 mg/L, p = 0.04 on day 15.

##### CRP in group A

In group A CRP-levels were elevated after intervention on day 1 and 3, and decreased thereafter comparable to the whole patient cohort.

##### CRP in group B

In group B CRP-levels showed a more pronounced elevation already at baseline and were significantly higher than in group A (69.1 ± 90.1 mg/L vs. 18.8 ± 14.5 mg/L; p = 0.012). CRP-values showed a further significant increase on day 3 (104.1 ± 90.3 mg/L, p = 0.049). Again, values decreased during the further course to a mean 42.4 ± 44.5 mg/L on day 15.

Laboratory values did not differ significantly between catheter- and needle-based interventions. Also there was no statistically significant difference between embolization with coils in combination with glue vs. glue alone.

##### Clinical course

Overall 13/60 patients (21.7%) experienced a febrile episode in the post-interventional period. There were no septic complications directly associated with transperitoneal access or embolization.

In group A no febrile episodes or other septic complications related to the intervention were observed during follow-up. Three non-septic post-interventional complications were recorded: aseptic edematous pancreatitis resolving on parenteral nutrition (n = 1; CTCAE grade 2); asymptomatic pulmonary glue embolization (n = 1; grade 1), upper extremity vein thrombosis resolving under heparinization (n = 1; grade 2). None of these complications prolonged hospitalization.

All 13 patients presenting with a febrile episode in the post-interventional period belonged to group B. These were attributable to concurrent.

- Pneumonia (n = 11), in 7 patients pneumonia was preexisting, in 4 cases it occurred during post-interventional follow-up,
- Cystitis (n = 1, post-interventional) or
- New inflammation at a drainage catheter already placed at the referring hospital (n = 1).

None of the episodes were attributed to the intervention itself. The other patients in group B already on antibiotics did not show febrile episodes after the intervention.

Three non-septic complications were observed in group B: asymptomatic pulmonary glue embolization (n = 2; grade 1); biliary peritonitis due to access through the gallbladder requiring cholecystectomy (n = 1; grade 4). In this patient leucocytes and CRP-values increased from 4.05 × 10^6^ cells/mL and 27 mg/L at baseline to 11.68 × 10^6^ cells/mL and 135 mg/L on day 10. After surgery on day 11 leucocytes and CRP decreased until day 15: 7.41 × 10^6^ cells/mL and 69 mg/L, respectively.

Both patients in whom antibiotics were started because of a transcolonic puncture remained asymptomatic. Leucocytes and CRP-values showed a maximum on day 1, without sustained or pathological elevation in both patients.

**Figure 5:**
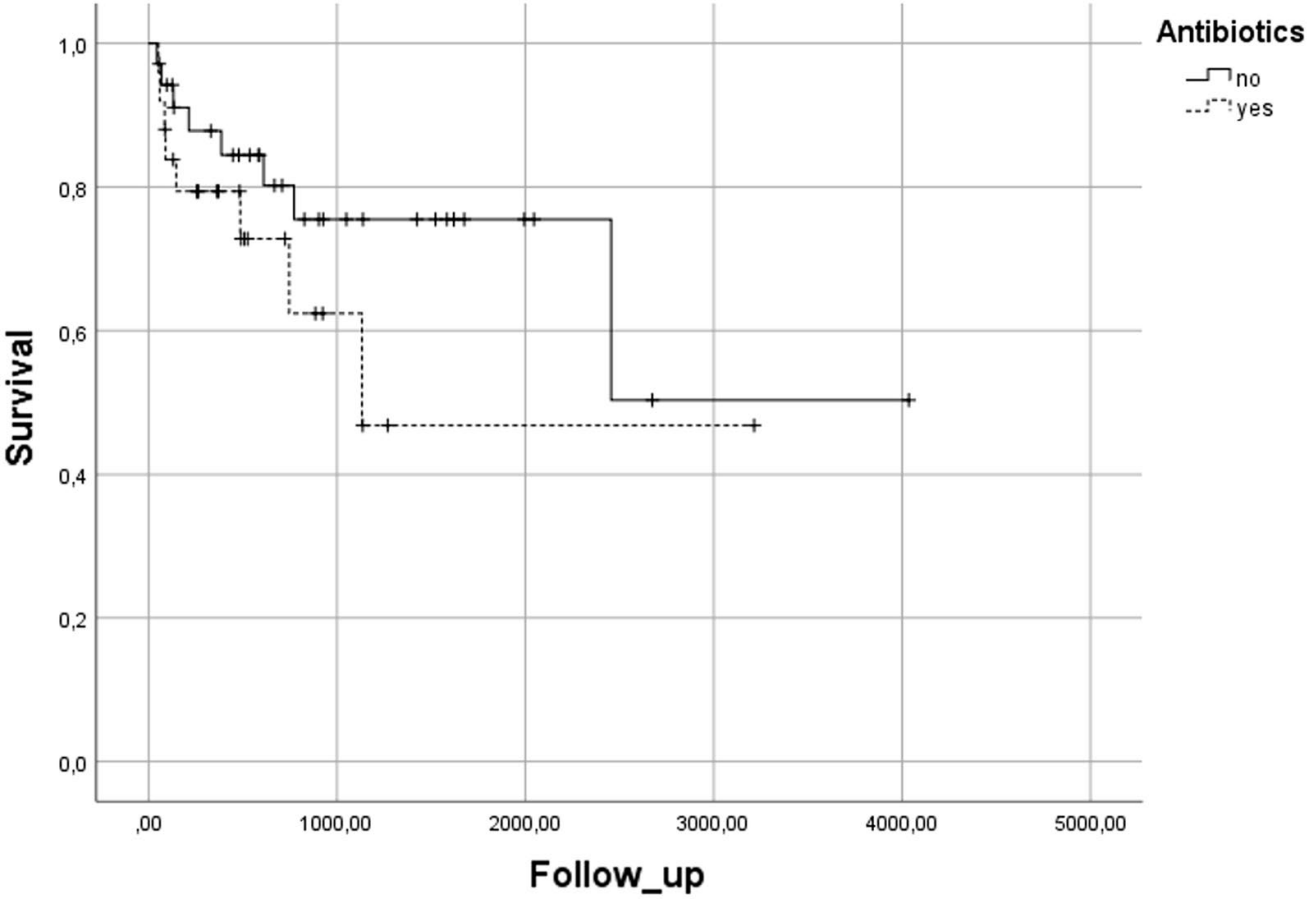
Kaplan–Meier plot demonstrating survival of patients with (group B) and without antibiotic use (group A).

There were no 30-day fatalities. 16/60 patients died during follow-up due to the underlying diseases. In group A 8/35 and in group B 8/25 patients died during follow-up. Median survival was 2558 (44–4036) days. There was no significant difference in median survival between groups (A: 2738 (44–4036) days vs. B: 1808 (53–3215) days; p = 0.222) (Fig. 5).

## Discussion

Lymphatic interventional procedures on the central lymphatic system often require a transabdominal access that is established by fine-needle puncture followed by introduction of a microcatheter^1,3,7^. As recently demonstrated^10^ the access path often transgresses intra-abdominal organs before entering the lymphatic system. This includes (potentially) colonized anatomical structures so that these interventions may be defined as “clean-contaminated” or even “contaminated”^18^. Antibiotic prophylaxis has been recommended to reduce the risks of infection from transintestinal access^9^ or from the skin flora^12^. However, so far no dedicated data on peri-interventional infectious complications, typical laboratory changes or peri-interventional antibiotic use are available.

The first question to be answered was how often procedure-related inflammatory complications are observed after transabdominal lymphatic interventions. Local inflammatory changes or abscesses were not seen in any patient. Also, septic complications were not observed. To the best of our knowledge, such problems have also not been published, underlining the overall safety of the procedure which is usually performed by experienced interventionalists. Lymphatic interventions have been performed at our institution for more than two decades without routine peri-interventional administration of antibiotics^7^. The majority of patients who did receive antibiotics in this study cohort did so for preexisting/concomitant pneumonias. Interestingly, septic complications associated with the intervention were observed neither in patients receiving antibiotics for other reasons nor in those without antibiotics. All febrile episodes in the post-interventional period occurred in patients receiving antibiotic therapy and could be explained by reasons unrelated to transabdominal access or embolization. As there were no clinically apparent inflammatory complications, the question arises, whether laboratory analysis of inflammatory markers showed changes after the interventions. Generally, an increase in leukocytes was seen on post-interventional day 1. This increase can be regarded as an expression of a non-specific post-operative/post-interventional reaction and/or a reaction to the embolization material - especially as values subsequently decreased and were significantly lower by day 10. For CRP it is well known from surgical studies that it also increases post-operatively. In uncomplicated cases, it usually reaches its maximum at about the third postoperative day, i.e. slightly later than leucocytes^19^. The increase is related to the extent of surgical trauma, and is smaller in minimally-invasive procedures^20,21^. In the absence of infectious complications CRP normalizes in the further clinical course^19^. In the patients in this study, the course of CRP was a similar one: it increased post-interventionally with a peak on day 3 and decreased thereafter. When comparing groups A and B, with regards to the course of leucocytes and CRP after intervention similar patterns emerged, even though there were higher absolute levels in group B. While in group A only 4/33 patients had a pre-interventional leukocytosis, 6/16 patients with pre-interventional antibiotics had pathologically elevated leucocytes. This difference can easily be explained by the patients’ poorer general condition with seven of them suffering from pneumonia. However, although there was a tendency towards a shorted survival in patients in need of antibiotic treatment, no statistically significant difference in overall survival was observed between both groups.

The results of this study demonstrate that even without routine peri-interventional antibiotic coverage, infectious complications of transabdominal lymphatic interventions are rare. If antibiotic coverage is forgotten at an institution usually employing routine antibiotics, our experience shows that this is not a cause for particular concern.

Notwithstanding the fact that patients at our institution do not routinely receive antibiotic coverage, there are indications in which such coverage should be provided (e.g. immunosuppression, valvular or congenital heart disease). Also, in case of recognized transcolonic access, antibiotic treatment starting immediately after the intervention is reasonable and seems sufficient. In the study cohort antibiotics were started peri-interventionally in only two patients because of a transcolonic access route. Duration of antibiotic treatment was about ten days in these two cases of colonic puncture. It is arguable that inadvertent bowel punctures may often go unrecognized during the intervention. Transcolonic puncture was recognized in the two patients because the colon was distended by air and therefore visible on fluoroscopy. Further bowel punctures in other patients cannot be excluded, but did not lead to any infectious complications possibly due to the very small gauge needles.

There are limitations of this study that have to be discussed. Clinical data were analyzed retrospectively with inherent methodological limitations. Especially under-reporting of (minor) complications must be considered. However, as patients included into the present study were under close clinical supervision for 10–15 days post-interventionally, it is unlikely that clinically relevant inflammatory complications have escaped detection. Prospective comparison of planned peri-interventional antibiotic prophylaxis and placebo would be necessary to analyze the treatment effects in detail. However, this was not the goal of this study and would be difficult to implement in the individualized treatment in this rare patient cohort. Also it might be argued, that the specific pre- and post-interventionally administered antibiotics might not be optimal to prevent/treat infectious complications of the procedure. Since we did not observe any inflammatory complications, this argument however is moot. Furthermore, it remains unknown whether the observed inflammatory reaction after an intervention is caused by the transabdominal access or by the foreign material introduced during embolization. A comparison of patients receiving transabdominal puncture but subsequent unsuccessful embolization (i.e. without embolization material) would be interesting. However, owing to a high technical success rate of embolization procedures at our institution, further analysis was not possible.

## Conclusion

Clinically relevant infectious complications directly associated with transabdominal lymphatic interventions are rare regardless of peri-interventional antibiotic treatment. Post-interventional elevations of leucocytes and CRP are observed in most patients after a lymphatic intervention with normalization of values over 10–15 days. As other infectious complications such as pneumonia may occur in this often multi-morbid patient cohort, targeted antibiotic therapy should be performed as clinically indicated.

## Data Availability

The datasets generated and/or analysed during the current study are not publicly available due to privacy protection guidelines of clinical data, but are available from the corresponding author on reasonable request.
